# There’s Something in What We Eat: An Overview on the Extraction Techniques and Chromatographic Analysis for PFAS Identification in Agri-Food Products

**DOI:** 10.3390/foods13071085

**Published:** 2024-04-01

**Authors:** Alessia Iannone, Fabiana Carriera, Sergio Passarella, Alessandra Fratianni, Pasquale Avino

**Affiliations:** 1 Department of Agriculture, Environmental and Food Sciences, University of Molise, Via De Sanctis, IT-86100 Campobasso, Italy; a.iannone2@studenti.unimol.it (A.I.); f.carriera@studenti.unimol.it (F.C.); sergio.passarella@studenti.unimol.it (S.P.); fratianni@unimol.it (A.F.); 2 Institute of Atmospheric Pollution Research, Division of Rome, c/o Ministry of Environment and Energy Security, Via Cristoforo Colombo 44, IT-00147 Rome, Italy

**Keywords:** PFAS, foods, toxicologic impact, human health, analytical chromatographic methods

## Abstract

Per- and polyfluorinated alkyl substances (PFASs) are a group of anthropogenic chemicals used in a range of industrial processes and consumer products. Recently, their ubiquitous presence in the environment as well as their toxicological effects in humans have gained relevant attention. Although the occurrence of PFASs is widely investigated in scientific community, the standardization of analytical method for all matrices still remains an important issue. In this review, we discussed extraction and detection methods in depth to evaluate the best procedures of PFAS identification in terms of analytical parameters (e.g., limits of detection (LODs), limits of quantification (LOQs), recoveries). Extraction approaches based on liquid–liquid extraction (LLE), alkaline digestion, and solid phase extraction (SPE), followed by liquid chromatography–mass spectrometry (LC-MS) and gas chromatography–mass spectrometry (GC-MS) analysis are the main analytical methods applied in the literature. The results showed detectable recoveries of PFOA and PFOS in meat, milk, vegetables, eggs products (90.6–101.2% and of 89.2–98.4%), and fish (96–108%). Furthermore, the low LOD and LOQ values obtained for meat (0.00592–0.01907 ng g^−1^; 0.050 ng g^−1^), milk (0.003–0.009 ng g^−1^; 0.010–0.027 ng g^−1^), fruit (0.002–0.009 ng g^−1^; 0.006–0.024 ng g^−1^), and fish (0.00369–0.017.33 ng g^−1^; 0.05 ng g^−1^) also confirmed the effectiveness of the recent quick, easy, cheap, effective, rugged, and safe method (QuEChERS) for simple, speedy, and sensitive ultra-trace PFAS analysis.

## 1. Introduction

PFASs are an emerging class of contaminants recently discussed by the scientific community. The widespread use of such substances for industrial and domestic purposes (stain and water-resistant textiles, food packaging, fire-retardant and fire-extinguishing products, pesticides, paints, personal care products, and surfactants) [1] is due to their chemical properties—in particular, to their excellent hydrophobic, oil phobic, and physicochemical stability [2]. These compounds are a group of synthetic organic fluoride compounds where hydrogen atoms are totally or partly replaced by fluorine atoms and connected with various functional groups (e.g., carboxylic and sulphonic acid groups) [3]. Owing to the strength of C-F bonds, they are persistent and hardly degradable in the environment [4]. After discharge, they accumulate in soil and tend to migrate to other places along with the water’s migration [2,5]. Humans can be exposed to PFASs through the ingestion of contaminated foods, air inhalation, dust ingestion, and dermal contact [6,7]. Several studies investigated the presence of PFASs in human blood [8,9,10,11,12,13,14]. Levels of perfluorooctanesulfonic acid (PFOS), perfluorooctanoic acid (PFOA), and perfluorohexanesulfonic acid (PFHxS) were found in blood samples collected from infants aged 3 months (1.48, 2.40, and 0.43 ng mL^−1^, respectively) [15] and human placental samples (0.242, 0.150, and 0.091 ng g^−1^) [16], confirming the PFAS transfer from mother to fetus [17,18,19]. A further route of PFAS exposure is related to the ingestion of indoor dust caused by the time that people spend in indoor environments (home and public spaces) [20]. Detectable concentrations of PFOS, PFOA, perfluorohexanoic acid (PFHxA), perfluorodecanoic acid (PFDcA), and perfluorododecanoic acid (PFDoDA) were found on air conditioner (A/C) filter dust sampled in various indoor microenvironments in Thessaloniki (Greece) in the range of 16–227 ng g^−1^; 3.6–72.5 ng g^−1^; 10–653 ng g^−1^; 3.2–7.4 ng g^−1^; and 3.8–13.1 ng g^−1^, respectively [21]. In addition, dust ingestion rates of 50–100 mg day^−1^ (toddlers) and 20–60 mg day^−1^ (adults) were obtained from the US. The EPA, in 2017 [22], found that while daily PFOA-equivalent intakes for toddlers were estimated at 1.3–1.5 ng kg^−1^ body weight (bw) d^−1^, exceeding the daily threshold derived from the European Food Safety Authority (EFSA) (0.63 ng kg^−1^ bw d^−1^) [23]. The higher ingestion of children compared to adults could be explained by the significant amount of time that children spent playing on the floor. Furthermore, their a tendency to place objects, including fingers, in their mouths significantly increased their exposure to compounds [24]. Several foods like fish [25,26], eggs [27], fruit and vegetables [28], meat, and dairy products [29,30] showed traces of PFASs. Due to the fact that people are exposed to PFASs through foods, some of them (i.e., PFOS, PFOA, PFNA, and PFHxS) have been subjected to European regulation. In particular, it was found that the highest concentrations of PFOA and PFOS (mean concentrations of 0.84 ng g^−1^ and 0.45 ng g^−1^, respectively) are in seafood, which contribute between 38 and 93% of the estimated dietary intake of the human population [31,32]. However, the widespread use of drinking water can contribute up to 75% of total exposure to PFASs [33], setting values at 0.1 μg L^−1^ for a sum of 20 individual perfluoroalkyl acids (PFAAs) (C4-C13 perfluoroalkyl carboxylic acid (PFCA) and C4-C13 perfluorosulfonic acid (PFSA)) and 0.5 μg L^−1^ for the total PFAS concentration [34,35]. To quantify the trace levels of these pollutants and understand their impacts on the environment, people, and living organisms, the development of accurate and robust analytical methods is required [36]. LC-MS and GC-MS are the most common techniques adopted for the qualitative and quantitative determination of PFASs in complex matrices [37,38]. In addition, for sample cleanup and extraction and best analysis of PFASs, a wide variety of approaches (e.g., liquid–liquid extraction (LLE), solid phase extraction (SPE), alkaline digestion) are necessary [39,40]. Finally, a quick, easy, cheap, effective, rugged, and safe method (QuEChERS) was developed to enhance the efficiency of extraction and reduce the cost and time of analysis, ensuring the enrichment of analytes in the sample and the remotion of the interferences, which could compromise the analysis [38]. In the present paper, we review the main extraction approaches and chromatographic techniques for PFAS determination in foods with the aim to identify the best protocols for their accurate identification and quantification at level traces. A literature search was conducted on the Scopus and Google Scholar databases considering papers published from 2015 to 2023. The selection strategy was based on the use of keywords, namely, “per- and polyfluoroalkyl substances”, “foods”, “toxicology”, “human health”, “extraction techniques”, and “chromatographic analysis”. Results obtained from literature search reported a total of 1530 articles: 378 articles were found using “per- AND polyfluoroalkyl AND substances” AND “foods” as keywords, 125 articles were found using “per- AND polyfluoroalkyl AND substances” AND “toxicology” as keywords, 952 articles were found using “per- AND polyfluoroalkyl AND substances” AND “human AND health” as keywords, 47 articles were found using “per- AND polyfluoroalkyl AND substances” AND “extraction AND techniques” as keywords, and 28 articles were found using “per- AND polyfluoroalkyl AND substances” AND “chromatographic AND analysis” as keywords. Finally, five validated documents available online were considered for toxicologic and legislative aspects.

## 2. Toxicology and Risk Assessment of PFASs

Several epidemiological and toxicological investigations have suggested adverse effects of PFASs on the reproductive, endocrine, and immunological systems [41]. Their adsorption and distribution throughout the human body could increase the risk of thyroid disease, blood, cholesterol, immune suppression, cancers of the kidney and testes, cardiovascular and kidney diseases, liver damage, neurological disruption, type II diabetes, osteoarthritis, and respiratory illness [42]. Among the PFASs, PFOA and PFOS are the most abundant perfluorinated compounds (PFCs) in the environment [43]. Their role as endocrine disruptors can lead to the neonatal mortality, neurotoxicity, nephrotoxicity, hepatotoxicity, immunotoxicity, and reproductive disorders [44,45]. For this reason, the World Health Organization (WHO), the United States Environmental Protection Agency (US-EPA), the California Office of Environmental Health Hazard Assessment (OEHHA), and the International Agency for Research on Cancer (IARC) have classified PFOA as a possible human carcinogen (group 2B) [46]. In addition, in 2021, the OEHHA added PFOS, its salts and transformation and degradation precursors to the list of chemicals known to the state to cause cancer for purposes of the Safe Drinking Water and Toxic Enforcement Act of 1986 (Proposition 65) [47]. Immunotoxicity of PFOA and PFOS was also confirmed by the U.S. National Toxicology Program (NTP, 2016). Based on the high level of evidence from studies of experimental animals and a moderate level of evidence from epidemiological studies, the NTP concluded that both PFOA and PFOS “are presumed to be an immune hazard to humans” because of the suppression of antibody responses to vaccines [48]. Thereafter, the U.S. Agency for Toxic Substances and Disease Registry (ATSDR, 2021) confirmed the association between decreased antibody response to vaccines and PFOA, PFOS, perfluorohexane sulfonic acid (PFHxS), and perfluorodecanoic acid (PFDA) serum concentration [49,50]. The same risk assessment for the sum of four PFASs (PFOA, perfluorononanoic acid (PFNA), PFHxS, and PFOS) was performed by the EFSA (2020) [51]. They listed “drinking water”, “fish meat”, “fruit and fruit products”, and “eggs and egg products” as the major sources of exposure to PFAS, identifying a lowest benchmark dose lower-bound confidence limit 10% (BMDL_10_) of 17.5 ng mL^−1^ for the sum of the four PFASs in serum of 1-year-old children. Since the significant accumulation over time and, consequently, health effects on the immune system, they recommended a total weekly intake (TWI) of 4.4 ng kg^−1^ bw [35].

## 3. Regulation

PFASs have gained significant public, scientific and regulatory attention due to widespread contamination and health disruptive effects [52,53]. In 2006, PFOS was the first compound to be regulated by the European Commission (EC) [54]. Consequently, many countries banned the use of PFOA, PFOS, and their precursors listed as persistent organic pollutants (POPs) under the Stockholm Convention (2009) [55]. In June 2022, the same Stockholm Convention parties also listed PFHxS, its salts and related compounds in Annex A to the Convention, setting them for elimination [56]. Recently, according to the opinion of the EFSA (2020), the EC amended Regulation 1881/2006 on the maximum levels for PFOS, PFOA, PFNA, and PFHxS in a selection of foodstuffs (egg, meat, fish, seafood, and offal) [57]. The next regulation, Regulation (UE) 2022/2388, entered into force on 1st January, established lowest limit for PFHxS at 0.20 μg kg^−1^ whereas the highest limit for PFOS is set at 35 and 50 μg kg^−1^ in meat and game offal, respectively [58,59]. Furthermore, in January 2023, the national authorities of Germany, Denmark, the Netherlands, Norway, and Sweden submitted a proposal to the European Chemicals Agency (ECHA) to restrict the use of PFASs under REACH Regulation. The objectives of the proposal support the ambitions of the EU Chemicals Strategy and the Zero Pollution Action Plan under the European Green Deal (European Commission, 2021) [60]. Since these strategies, the European Commission and European countries have additionally co-founded the European Human Biomonitoring Initiative (HBM4EU) under Horizon 2020 [61]. The aim of the initiative is to assess the actual exposure of citizens to environmental chemicals, including PFASs, and their possible health effects in order to support policy makers’ efforts to ensure chemical safety and to improve health in Europe [62,63].

## 4. PFAS Analysis on Food Matrices

### 4.1. Meat

Meat and meat products are generally included in human diets for the supply of numerous nutrients (i.e., protein, iron, vitamins, essential amino acids) [64,65]. However, their frequent consumption needs to be controlled for possible PFAS contamination. Ultra-low traces of PFASs (ug g^−1^ or pg g^−1^) in complex matrices such as meat require a preliminary phase based on sample preparation and/or pretreatment before chromatographic separation. Table 1 summarizes the extraction techniques (e.g., SPE, solid liquid extraction (SLE), alkaline digestion) investigated by several authors. The following PFAS analysis by LC, HPLC, or GC is performed using ESI-MS/MS and a high-resolution mass spectrometer (HRMS) [66]. Moreover, the UPLC-MS/MS method based on the use of small particle sizes of stationary phases (such as 1.7 μm) and high chromatographic column pressure is also employed to ensure the resolution and separation of PFASs [67,68]. In the study of Rawn et al. [40], they developed a method for the determination of 21 PFASs in different food matrices including chicken nuggets. They carried out the extraction of compounds using ACN as an extraction solvent added to a sample contained in a polypropylene (PP) centrifuge tube. This solvent resulted in better recoveries (Table 1) compared to other solvents tested (e.g., MeOH, tetrahydrofuran (THF)). They used Oasis SPE-WAX cartridges for the cleaning and the removal of interference species followed by the quantification of PFASs through an LC-ESI (-)-MS/MS system. Sadia et al. [69] tested different digestion methods (acid or alkaline), extraction such as in the ion pair method (IPM), or SLE and organic solvents (MeOH, ACN). The aim of their study was the development and validation of a sensitive analytical method for the determination of PFOS, PFOA, and PFHxS in chicken meat and beef. Due to the better recoveries (>70%), alkaline digestion and SLE with ACN as extraction solvent were chosen for the method’s optimization. The further clean-up of food samples was conducted using SPE-WAX (6 mL, 150 mg, 30 µM) cartridge. The instrumental analysis of contaminants was performed by LC-MS/MS analysis by Waters Acquity UPLC coupled with XEVO TQS mass spectrometer in ESI (-) mode.

In the last few years, several authors applied the QuEChERS method for the analysis of ultra-traces level of pesticides, veterinary drugs, PFAS and other organic compounds in food matrices [39]. The advantage of this technique is due the speed and the simplicity of two steps of analysis based on a first ACN salting-out extraction (SOE) followed by dispersive solid phase extraction (d-SPE) to clean up and to remove matrix interferences [70]. Chiesa et al. [64] investigated the distribution of PFAS and polybrominated diphenyl ether (PBDE) trace contaminants in pork from eight different European countries (Austria, Denmark, French, Germany, the Netherlands, Italy, Poland, and Spain). Extraction of PFASs and PBDEs was carried out using the SPE-WAX and QuEChERS methods, respectively. Both methods showed high specificity and selectivity for each compound analyzed. In addition, the results obtained through liquid chromatography coupled with high-resolution mass spectrometry (LC-HRMS) and GC-MS/MS showed that no PFASs were detected in pork samples except PFOA in only one Australian sample at a concentration of 0.531 ng g^−1^. PBDEs were detected in 3 out of 77 samples: one coming from Germany (0.53–0.77 ng g^−1^) and two coming from the Netherlands and Italy (0.53 ng g^−1^ and 0.62 ng g^−1^, respectively).

Gallocchio et al. [70] developed and validated a fast and sensitive method for the simultaneous analysis of 14 PFASs (both short chain (SC) and long chain (LC)), including Gen X, C6O4, PFOA, PFOS, PFNA, and PFHXs in different foods of animal origin, (i.e., meat, liver, egg, and milk). For this purpose, they employed the QuEChERS extraction and cleanup method combined with LC-MS/MS. Compared to the classical cleanup method based on SPE-WAX validated by the same authors in another published study [71], the advantage of the QuEChERS procedure was related to shortened analysis duration. The validated method showed good linearity (Pearson’s R > 0.99) with very low LODs (7.78–16.35 ng kg^−1^, 8.26–34.01 ng kg^−1^, 6.70–33.65 ng kg^−1^, and 5.92–19.07 ng kg^−1^ for milk, liver, egg, and muscle, respectively) and appropriate LOQs (50 ng kg^−1^ for all compounds except for GenX and C_6_O_4_, which were 100 ng kg^−1^). Similarly, Genualdi et al. [72] developed a QuEChERS method for the extraction of 16 PFASs in total diet study (TDS) samples analyzed by LC-MS/MS. The QuEChERS method validated was similar to what was reported in a previous paper: 5 g of the sample was placed in a 50 mL conical PP centrifuge tube fortified with a mass-labeled PFAS mixture. Then, 10–15 mL of water and 10 mL of ACN were added along with 150 μL of FA. The samples were vortexed for 1 min and then, for inducing phase separation and contaminant partitioning in organic phase, extraction salts were added (ECMSSCFS-MP with 6000 mg of magnesium sulphate (MgSO_4_) and 1500 mg of sodium chloride (NaCl)) to increase the ionic strength of the solution. The samples were shaken again for 5 min using a digital pulse mixer/vortexer at 1500 rpm and centrifuged for 5 min at 10,000 relative centrifugal force (rcf). All the extracts were transferred into a prefilled centrifuge tube with a d-SPE sorbent (ECMPSCB-MP with 900 mg of MgSO_4_, 300 mg of primary–secondary amine (PSA), and 150 mg of graphitized carbon black (GCB), UCT, Bristol, PA, USA), shaken, and centrifuged using the same condition as the salt tubes. Finally, 5 mL of the extract was filtered through a 0.2 μm nylon filter, and the ISTD (d5-NEtFOSAA) was added. During method optimization, SPE cartridges (WAX SPE Cleanup, Push-Thru WAX SPE Cleanup) were also investigated. Results showed that the use of the push-through SPE cartridge allowed for a rapid cleanup option for food samples compared to the WAX SPE cleanup cartridge. Furthermore, of the 179 samples analyzed for determining the PFAS concentrations, only 3 samples showed concentrations above the MDLs. As a matter of fact, only 83 and 87 ng kg^−1^ PFOS in two tilapia samples and 86 ng kg^−1^ PFOS in ground turkey were measured. A QuEChERSER (quick, easy, cheap, effective, rugged, safe, efficient, and robust) sample preparation mega-method was validated by Taylor and Sapozhnikova [67] for determining PFASs in food. This method was able to monitor chemicals over a broad range of physicochemical properties. The QuEChERSER protocols can be summarized as follows: 5 mL of 4:1 (*v*/*v*) ACN/water was added to 1 g tissue samples spiked with standard and ISTD mixtures. Samples were vortexed, shaken for 10 min on a platform mixer, centrifuged at 3711 rcf for 3 min, and then 0.2 mL of supernatant was transferred to a PP micro-centrifuge tube in duplicate. Once the supernatant evaporated to near dryness with N_2_ gas, 0.8 mL of MeOH or initial mobile phase (95:5 H_2_O: MeOH) was added. Samples were vortexed, centrifuged at 12,500 rcf for 5 min, and then the supernatant was transferred to a PP autosampler vial for analysis (LC portion). For instrument top sample preparation (ITSP) cleanup (GC portion), the ACN layer was transferred to an autosampler vial. To compare cleanup efficiency and evaluate PFAS recovery, the GC portion was an analyzed by GC-MS and LC-MS, respectively. A comparison of recoveries, matrix effects, and cleanup efficiency between the QuEChERSER method validated and the US Food and Drug Administration (FDA) and USDA Food Safety and Inspection Service (FSIS) PFAS extraction methods was also reported by the authors. The results showed that the FDA and QuEChERSER methods performed better than the FSIS method, especially in beef and catfish matrices, with acceptable recoveries (106 ± 14% vs. 88 ± 10% in beef; 101 ± 14% vs. 84 ± 11% in catfish) in both cases. However, all three methods reported average absolute and relative matrix effects within ±20%. Finally, additional validation was performed to compare instrumental performance of the Q-Orbitrap high-resolution mass spectrometry (HRMS) and QqQ low-resolution mass spectrometry (LRMS) systems. The analysis by means of HRMS achieved acceptable recoveries (70–120%) and relative standard deviations (RSDs) ≤20% for all 33 target analytes at the 1 and 5 ng g^−1^ levels and 67–88% of analytes at the 0.1 ng g^−1^ level. Similar results were shown by QqQ method. Compared to the HRSM method, this approach provided better recovery at 0.1 ng g^−1^ level (70–91%), but more variability spiking levels at 1 and 5 ng g^−1^ were detected. Despite better detectability, the HRMS method showed lower LODs and LOQs than the QqQ method (Table 1). The difference in LOQs was most pronounced for pork, but all matrices showed lower medians and less variance in HRMS-derived LOQs. Recently, a nano-structural and/or nano-functionalized solid phase microextraction (SPME) technology was proposed for food, bioanalytical, and clinical analysis due to their simplicity and operation-friendly properties. However, the performance of nano-functionalized SPME also depends on the specific sorption and desorption properties of adsorbent coated on supports. Based on these assumptions, Li et al. [73] realized a fluorinated boron nitride nanosheet (F-BNN)-coated fiber for the analysis of trace PFASs from food samples. The F-BNNs were fluorinated with a hydrothermal reaction and used as coasting of SPME fiber for sensitive analysis of PFAS contamination. The protocol of DI-SPME employed in the study was as follows: first, the SPME fiber was immersed into a 20 mL of sample solution to adsorb PFAAs and after an equilibrium for 20 min at 50 °C, the SPME fiber was withdrawn and washed with 5 mL of pure water. Desorption of PFAA analytes was carried out with 3 mL ACN by agitation. Thus, the eluents were filtered through a 0.22 μm syringe filter and evaporated to dry under a gentle nitrogen (N_2_) stream. Finally, for instrumental analysis, the adsorption and re-dissolution of PFAAs with 100 μL of MeOH/water (10%, *v*/*v*) were performed. Results obtained by the combination of SPME with HPLC-MS/MS showed low MDLs (Table 1) with satisfactory repeatability (RSD% < 13.5%). Dominant PFAAs such as PFOS and PFOA were detected in meat samples with concentrations ranging from 35.4 to 452.3 pg g^−1^. On the other hand, PFOA, PFNA, and PFHxS showed concentrations ranging from 79.9 to 209.5 pg g^−1^. Consequently, this homemade SPME fiber could be used for routine monitoring of PFAAs in different foodstuffs. Following Li’s protocol [73], Sungur et al. [74] detected the highest PFOA concentrations in cow meat (5.15 ng g^−1^), cow kidney (5.65 ng g^−1^), cow spleen (5.06 ng g^−1^), and chicken liver (5.02 ng g^−1^). The authors evaluated the presence PFOA and PFOS in food and beverages sold in Turkey, by means of LC-MS/MS. Preparation and extraction of samples involved an initial homogenization of 1 g of sample with 5 mL of high-purity Milli-Q water, followed by the addition of 1 mL of 0.5 M tetrabutylammonium hydrogen sulphate solution (TBAHS) and 2 mL of sodium carbonate (Na_2_CO_3_) buffer (0.25 M, pH 10) to 1 mL of the homogenate sample. Moreover, 5 mL of MTBE was added to the prepared mixture and shaken for 20 min. After the separation of the organic and aqueous layers by centrifugation, an exact volume of MTBE (4 mL) was removed from the solution. A double separation of aqueous mixture rinsed with MTBE was realized, and both the rinses were combined in a second PP tube. The solvent was evaporated under N_2_ and replaced with 0.5 mL of MeOH. The final extract was passed through a nylon mesh filter (0.2 μm) into an HPLC vial for PFOA and PFOS analysis. Based on the results obtained, the high PFOA values detected in meat samples could be related to the migration of PFASs from herbivorous animals that feed on contaminated plants and water to animal products available commercially and consumed by people. PFOS contents were also found in samples of meat (0.841 ng g^−1^). As matter of fact, the authors concluded that meat and offal were the major sources of PFOA intake, unlike fish, where it was found that the highest PFOS concentrations were in the range of 7.96 to 52.43 ng g^−1^ compared to PFOA levels, ranging between 0.147 and 0.543 ng g^−1^.

**Table 1:** Summary of analytical procedure for analysis of PFAS in meat products and their recoveries (expressed as%), LODs (ng g^−1^), and LOQs (ng g^−1^). N/A = not available; ^a^ = MDL.

Matrix	Extraction and Pretreatment	Analysis	Recoveries	LOD	LOQ	Ref.
Pork	SPE-WAX for PFAS extraction QuEChERS method for PBDE extraction	LC-HRMS for PFAS analysis, GC-MS/MS for PBDE analysis	80–119 for PFAS, 88–93 for PBDE	0.005–0.050 for PFAS	0.015–0.150 for PFAS	[64]
Bovine and swine muscle, bovine liver	QuEChERS method	LC-MS/MS	80–120	0.00826–0.03401 for liver, 0.00592–0.01907 for muscle	0.050 except for GenX and C_6_O_4_ with 0.100	[70]
Chicken nuggets, beef steak, ground beef, chicken leg, pork bacon, pork chop, pork sausage, chicken breast, cured ham, sausage/salami combination, frankfurter (beef/pork), lamb chop, turkey breast, and ground turkey	QuEChERS method	LC-MS/MS	40–120	0.086 PFOS in ground turkey	N/A	[72]
Beef, pork	DI-SPME with F-BNN-coated fiber	HPLC-MS/MS	77.7–110.5	0.0036–0.0158 ^a^	N/A	[73]
Chicken nuggets	Oasis SPE-WAX cleanup and extraction with ACN solvent	LC-MS/MS	42–221	0.006 for perfluorodecane sulfonate (L-PFDS), 1.76 for perfluorohexyl ethanoic acid (FHEA)	0.018 for L-PFDS, 5.28 for FHEA	[40]
Chicken meat, beef	Alkaline digestion, SLE with ACN solvent followed by SPE-WAX cleanup	LC-MS/MS	77–89	0.0011, 0.0014 and 0.0016 for PFHxS, PFOA and L-PFOS	0.0031, 0.0034 and 0.0049 for PFHxS, PFOA and L-PFOS	[69]
Cow meat (heart, kidney, spleen, liver); sheep meat (heart, kidney, spleen, liver); chicken meat (liver, heart, gizzard)	Extraction with MTBE solvent	LC-MS/MS	90.6–101.2 for PFOA and 89.2–98.4 for PFOS	0.038 for PFOA and 0.002 for PFOS	0.125 for PFOA and 0.007 for PFOS	[74]
Beef, chicken, pork	QuEChERSER method	Ultra-performance liquid chromatography–high resolution mass spectrometry (UPLC-HRMS), ultra-high performance liquid chromatography–tandem mass spectrometry (UHPLC-MS/MS) with QqQ	88 ± 10	HRMS Method: Beef (0.0009–0.267), chicken (0.0006–0.14879), pork (0.0046–0.3026) QqQ Method: Beef (0.006–0.048), chicken (0.003–0.078), pork (0.020–0.228)	HRMS Method: Beef (0.0027–0.8091), chicken (0.0018–0.4506), pork (0.014–0.9169) QqQ Method: Beef (0.018–0.145), chicken (0.009–0.237), pork (0.061–0.689)	[67]

#### Overall Summary

From the literature research, it emerged that several approaches have been applied for PFAS extraction. The push-through SPE cartridge allowed for faster and cleaner isolation of analytes than OASIS-SPE-WAX. However, QuEChERSER methods determined higher recoveries of PFASs (on average <101%). For the chemical analysis, the use of QqQ could be suggested due to its sensitivity (e.g., lower LOD and LOQ values than other described techniques).

### 4.2. Milk and Dairy Products

People are exposed to PFASs through milk and dairy products [39,75,76,77]. Their presence in milk matrices is due to the high affinity to bind proteins, particularly β-lactoglobulin, and their tendency to concentrate along the trophic chain [78]. Other factors that could be contribute to PFAS contamination include health of the animal, hygiene of the staff, safety level of the animal’s feed, and contamination of the final product originating from processing and packaging [77,79]. Due to the large quantity of proteins and lipids in milk, a careful sample pretreatment before analysis is necessary in order to reduce matrix artefacts and enhance PFAS detection and quantification. Table 2 summarizes the main approaches developed by several authors to extract and detect PFASs in milk products. For example, Berendsen et al. validated an analytical method for analysis of 13 PFASs including PFCAs, PFSAs, and HFPO-DA in milk and eggs according to the Dutch validation standard NEN 7777:2011 (NEN 2011) [75]. Extraction was conducted using 10 mL lead acetate (Pb(OAc)_2_) solution, 10 mL MeOH, and 100 μL FA added to a sample previously fortified with 25 μL of standard solution. The centrifugated extract (10 min at 3500 rcf at 2 °C) was purified by means of SPE through a Strata-X-AW cartridge and eluted with 5 mL 2% AH in ACN. The following UHPLC-MS/MS PFAS analysis confirmed the suitability of the method to quantify all compounds in milk samples with accuracy ranging from 87% to 113%. The relative within-laboratory reproducibility (RSDRL) ranged from 12% to 39%, repeatability (RDSr) ranged from 6% to 18%, except for PFDS (85% and 60% for RSDRL and RDSr, respectively), which showed poor accuracy and high uncertainly. Further, low LOQs were achieved for all analytes, but for PFOA, the determined LOQ was slightly higher than required (0.005 ng g^−1^ instead of 0.003 ng g^−1^). A sensitive method for determination of 27 PFASs in raw and processed milk samples from 13 farms across the United States (U.S.) was applied by Hill et al. [79]. To evaluate the efficiency of SLE, the authors used two different extraction solvents and cleanup procedures: (i) 0.1% FA in MeOH followed by cleanup with only ENVI-Carb cartridges, (ii) 0.1% FA in MeOH extraction followed by cleanup with Oasis WAX resin-loaded atop of ENVI-Carb cartridges, and (iii) 0.1% AH in MeOH followed by cleanup with Oasis WAX powder-loaded atop of ENVI-Carb cartridges. Results achieved by the combination of solvent extraction and ENVI-Carb cleanup cartridge followed by HPLC-MS/MS showed high recoveries and better MDLs for a large part of the PFASs analyzed (Table 2). PFOA and PFOS are of the two most frequently reported compounds in milk and dairy products due the ability of cows both to bioaccumulate and to biotransfer PFASs from plasma into milk. Furthermore, industrial food processing and packaging can also influence PFAS contamination in milk [78,79]. Xing et al. [78] measured the levels of PFOA and PFOS in milk and yogurt in Xinjiang (China). They extracted the chemicals by means of ultrasonic extraction methanol (UE-MeOH), then purified them using SPE with an HLB cartridge. The analysis by HPLC-MS/MS reported higher detection rates and mean concentrations of PFOS in both retail milk (39.6% and 24.5 ng L^−1^, respectively) and yogurt (48.1% and 31.8 ng L^−1^, respectively) than PFOA (33.0% and 16.2 ng L^−1^ (milk), 37.0% and 22.6 ng L^−1^ (yogurt), respectively). This condition could be dependent both on the greater ability of the animal to accumulate PFOS than PFOA and on high exposure to PFASs in the industrialized and populated areas of northern Xinjiang. The occurrence of PFASs in foodstuffs and their migration from food contact material was also investigated by Huang et al. [80]. The authors evaluated the PFAS concentration in ice cream, bubble tea, and instant noodles, typical fast foods mainly consumed among children and young people. Extraction of PFASs was performed as follows: an aliquot of samples (2 mL) was spiked with five isotope-labeled surrogate standards (2 ng mL^−1^), vortexed (2 min), and centrifugated (9600 rpm for 20 min) after the addition of 6 mL ACN. The supernatant was collected and another 6 mL of ACN was added to the remaining residue. After the second extraction, the supernatants were combined and blown under N_2_ gas to about 0.8 mL at 40 °C, reconstituted with ultrapure water (8 mL), and centrifuged at 9600 rpm for 20 min. The further cleanup and concentration of the supernatant were carried out by the use of OASIS WAX-SPE cartridges. The target compounds were eluted with 4 mL of 9% ammonia in MeOH. The extracts were concentrated to 50 μL under a gentle stream of N_2_ at 60 °C, reconstituted in 200 μL of MeOH/water (50:50, *v*/*v*), and filtered through a 0.22 μm membrane before UHPLC−HRMS analysis. The findings of the paper, based on analyzing of 27 PFASs, suggested that the intake of the foods investigated could be a source of PFAS exposure. As matter of fact, all samples analyzed showed some PFASs; particularly, a high concentration of PFOA, PFBA, and 6:2 polyfluoroalkyl phosphate monoester (PAP) was observed. The highest abundance of PFOA (1.16–4.38 ng g^−1^), found in the ice cream samples, confirmed the possible presence of PFASs in dairy products. This compound, commonly used in manufacturing industries, may migrate from the packaging material and the production processes to the ice cream, for which the main components are milk and cream. However, high frequencies of PFOA, PFBA, 6:2 PAP, and 6:2 diPAP were also detected in the ice cream samples. Based on the limits of SPE, several authors applied the QuEChERS method as an alternative extraction cleanup procedure.

Macheka et al. [81] analyzed the occurrence of PFASs in dairy milk and infant formulas. The QuEChERS method validated involved the addition of 10 mL of ACN and 10 mL of deionized water to 1 mL of sample spiked with 2 ng mL^−1^ of ISTD (_13_C PFOA and _13_C PFNA). This was followed by the addition of 4 g MgSO_4_ and 1 g NaCl salts dissolved through vigorous agitation of the mixture through vortexing (30 s) and centrifugation (8000 rpm for 10 min set at 4 °C). Before the UHPLC-MS/MS analysis, 200 µL of the supernatant was withdrawn and filtered through a 0.22 μm nylon membrane filter into a 1.5 mL LC vial. Results of the study showed that the concentration of Σ15 PFASs in dairy milk and infant formula ranged 0.08–15.1 ng mL^−1^ and 0.42–5.74 ng mL^−1^, respectively. In both matrices, the most prevalent PFASs were PFBA, perfluoropentanoic acid (PFPeA), perfluoroundecanoic acid (PFUnDA), perfluorotridecanoic acid (PFTrDA), and PFDoA with detection frequencies >96%. The highest PFDoA concentrations (2.02 ng mL^−1^ and 2.76 ng mL^−1^) were also reported in infant formulas and dairy milk. The analogue distribution profile of PFASs obtained suggested similar sources of contamination except for PFOS, where the concentrations in infant formula were significantly higher than in dairy milk. Yu et al. [82] developed a highly sensitive and reliable method for determination of 20 PFASs in milk using the QuEChERS approach and online interference trapping LC-MS/MS. The protocol included a first extraction of PFASs from a spiked milk sample (1.0 ng for each standard compounds) with 10.0 mL of ACN and 30 uL of concentrated hydrochloric acid (HCl) followed by cleanup of the extract transferred to a 15 mL low-density polyethylene (LDPE) centrifuge tube containing 60 mg of PSA, 40 mg of C_18_, and 10 mg of GCB. By analyzing a series of calibration solutions containing various amounts of standard compounds and fixed amounts of isotope-labeled ISTD, the authors showed that the method reported very wide dynamic linear ranges for all PFASs analyzed, with PFCAs in the range of 0.010–20.0 µg L^−1^ and PFSAs in the range of 0.050–20.0 µg L^−1^. The level of PFAS contamination was also evaluated by Sznajder-Katarzyńska et al. [77]. The authors investigated the presence of 10 PFASs in thirty-five different foods including milk, cottage cheese, natural yoghurt, kefir (bonny clabber), sour cream, Camembert-type cheese, and butter purchased from Polish markets in 2017. Samples were analyzed using a QuEChERS method with the d-SPE sample cleanup previously validated by Surma et al. [83] followed by micro-HPLC-MS/MS analysis. The protocol included a first extraction processes of 10 g or 10 mL of each sample spiked with 10 mL ISTD solution (2.5 µg mL^−1^) with 10 mL ACN and 150 mL FA by sonication and mechanical agitation for 2.5 min and 1 min. Then, 1 g NaCl and 4 g MgSO_4_ were added to the tube, and it was shaken vigorously for 1 min. After the separation phase by centrifugation (at 15 min at 8693 rcf), 6 mL of ACN was added to a PP 15 mL tube containing 0.15 g ENV SPE Bulk Sorbent and 0.90 g MgSO_4_. The tubes were shaken again for 30 s and centrifugated for 5 min at 8693 rcf. To remove residual fat, 4 mL of supernatant was placed into a 4 mL tube stored at −12 °C for 20 h. In the next step, samples were filtered through a paper filter and evaporated to dryness in a vacuum concentrator at 40 °C. Finally, before micro-HPLC-MS/MS analysis, the residues were dissolved in 1 mL MeOH and subsequently diluted fivefold in water via addition of 1% (*v*/*v*) FA. The results shown in this paper confirmed the high applicability of the QuEChERS method and micro-HPLC-MS/MS for PFCA and PFSA detection in milk and milk products. All PFASs were detected with RSDs lower than 10%. PFPeA was the compound least frequently identified in the group of milk and milk products with lowest total concentration of 0.43 ng g^−1^, whereas the highest total concentration of PFBuS was found at 13.34 ng g^−1^. The PFOS concentrations were in the range of 0.02–0.47 ng g^−1^, while a not very high concentration of PFOA was identified (ranging from 0.05 ng g^−1^ for butter to 0.50 ng g^−1^ for Camembert-type cheese). In addition, the use of polymer-based sorbent ENV allowed for efficient PFAS extraction from milk matrices in terms of recoveries, LODs and LOQs (Table 2). The extraction procedures and instrumental analysis of PFASs developed by Li et al. [73], Genualdi et al. [72], Sadia et al. [69], Gallocchio et al. [70], and Sungur et al. [74] are largely described in Section 4.1. The use of F-BNN@SPME fiber on sensing trace PFAAs exhibited high performance with low MDLs (Table 2) for milk. Only PFOS was detected in raw milk, with a concentration of 37.9 pg mL^−1^, whereas PFPeA, PFOA, PFHxS, and PFOS were found in bagged milks, with concentrations from 21.2 to 145.6 pg mL^−1^ [73]. Low LODs and appropriate LOQs with recoveries up to 120% were achieved by using the QuEChERS method [70,72]. In addition, trace levels of PFOA and PFOS with LODs ranging between pg L^−1^ and ng L^−1^ were also detected in milk using different extraction procedures (alkaline digestion, SLE, LLE) [69,74].

Several studies have also confirmed the presence of PFASs in human breastmilk. It has been reported that placental transfer and breastfeeding are both potential pathways to PFAS exposure for nursing infants. The transfer mechanism from maternal serum to milk is regulated by the binding affinities of PFASs to serum protein [24,84]. Serrano et al. [85] applied UHPLC-MS/MS to evaluate the concentration of eleven PFASs in pooled milk samples collected from 82 donor mothers at the Human Milk Bank of the Virgen de las Nieves University Hospital (Granada, Spain). Sample treatment was performed using salting-out-assisted liquid–liquid extraction (SALLE) carried out by addition of salt mixture (600 mg of NaCl, 200 mg di-sodium hydrogen citrate (Na_2_HCit), and 200 mg tri-sodium citrate (TSC)), vortexed, and centrifuged at 4000 rpm for 10 min. The resulting supernatant was concentrated to 1 mL under N_2_ stream and added with 10 mL (10% NaCl aqueous solution (*w*/*v*) at pH 2) and 1.5 mL of a mixture of trichloromethane (TCM)/ACE 4:1 (*v*/*v*). The mixture obtained was shaken and centrifuged for 5 min at 4000 rpm. After the organic phase evaporated, the final residue was dissolved with 100 µL of a mixture of 5 mM ammonium acetate (NH_4_OAc) (pH 4.5) and ACN 30:70 (*v*/*v*) before the analysis. Detection frequencies of PFASs were shown in 24–100% of breastmilk samples. Perfluoroheptanoate (PFHpA) was detected in all samples, followed by PFOA (84%), PFNA (71%), PFHxA (66%), and PFTrDA (62%), whereas the remaining compounds were detected in less than 40% of samples. Finally, the median of the sum of PFAS concentrations was 87.67 ng L^−1^ and was higher for SC than LC PFASs. Amziane et al. [24] developed an ultra-high performance supercritical fluid chromatography coupled with tandem mass spectrometry (UHPSFC-MS/MS) method for the analysis of PFASs in food matrices and breastmilk. Samples collected were extracted by 15 mL of a 0.01 M potassium hydroxide methanolic (KOH/MeOH) solution. Following this, two SPE purifications were performed using Oasis WAX SPE and ENVI Carb SPE cartridges eluted with a mixture of MeOH/NH_4_OH 32% (99.5/0.5; *v*/*v*) and a mixture of MeOH/glacial acetic acid (GAA) (80/1; *v*/*v*), respectively. The results obtained showed that the sum of PFASs detected in breastmilk was less than in other matrices (1.07 ng g^−1^ fresh weight (fw) vs 1.35 ng g^−1^ fw in chicken eggs, 1.48 ng g^−1^ fw in poultry meats, 1.99 ng g^−1^ fw in red meats, and 8.05 ng g^−1^ fw in fish), with a low LOQ for 97% PFASs [16]. Finally, a UHPLC-MS/MS for a sensitive determination of PFASs in breastmilk, retail milk, and infant formulas was developed by Abafe et al. [39]. They investigated the effectiveness of three different extraction techniques: LLE, SPE, and a simplified QuEChERS procedure. Comparable results from the most of the PFASs targeted were obtained for both LLE and the simplified QuEChERS extracts. However, the simplified QuEChERS procedure was optimized and applied, showing suitability for the sensitive analysis of C_4_-C_14_ PFASs. All target PFASs showed determination coefficient (R^2^) values ranging between 0.9843 and 0.9998. Among these, PFDA, PFUnDA, PFDoA, and PFTrDA were mainly detected in retail milk, while PFBA, PFPeA, PFBuS, and PFHxA were more prevalent in infant formula and breastmilk.

**Table 2:** Summary of analytical procedure for the analysis of PFASs in milk and dairy products and their recoveries (expressed as%), LODs (ng g^−1^), and LOQs (ng g^−1^). N/A = not available; ^a^ = decision limit (CCα); ^b^ = detection capability (CCβ); ^c^ = method detection limit (MDL); ^d^ = ng g^−1^.

Matrix	Extraction and Pretreatment	Analysis	Recoveries	LOD	LOQ	Ref.
Breastmilk, retail dairy milk, and infant formulas	QuEChERS method	UHPLC-MS/MS	65–136	0.03–0.05 ^a^ 0.04–0.1 ^b^	0.005–0.050	[39]
Human breastmilk	Extraction with KOH/MeOH solution followed by two SPE purification with WAX SPE and ENVI-Carb SPE cartridges	UHPSFC-MS/MS	N/A	N/A	<0.2 except for perfluorodecyl ethanoic acid (FDEA) (1 ng g^−1^)	[24]
Milk	Extraction with Pb(OAc)_2_ solution, MeOH and FA followed by SPE cleanup with Strata-X-AW cartridge	UHPLC-MS/MS	N/A	0.0025–0.75	0.005–0.1	[75]
Cow milk	QuEChERS method	LC-MS/MS	91.3–121.8	0.00778–0.01635	0.05 for all compounds except for GenX and C_6_O_4_ (0.1 ng g^−1^)	[70]
Milk	QuEChERS method	LC-MS/MS	40–120	0.007–0.042 ^c^	N/A	[72]
Raw and processed milk	SLE with FA in MeOH and AH in MeOH followed by SPE cleanup with ENVI-Carb and Oasis WAX cartridges	HPLC-MS/MS	70–141	0.8–22 ^d^ for all PFAS except 144 ^d^ for PFBA	N/A	[79]
Ice cream	Ultrasonic-assisted extraction (UAE) with ACN solvent followed by SPE cleanup with Oasis WAX cartridge	UHPLC-Orbitrap HRMS	52–107	0.001–0.009	0.002–0.020	[69]
Raw milk and bagged milk	DI-SPME with F-BNN-coated fiber	HPLC-MS/MS	85–110	0.9–3.9 ^c,d^	N/A	[73]
Dairy milk and infant formula	QuEChERS method	UHPLC-MS/MS	93–120	5–50 ^d^	5–50 ^d^	[81]
Cow milk and butter	Alkaline digestion, SLE with ACN solvent followed by SPE-WAX cleanup	LC-MS/MS	93–101	0.0011, 0.0014 and 0.0016 for PFHxS, PFOA, and L-PFOS	0.0031, 0.0034 and 0.0049 for PFHxS, PFOA, and L-PFOS	[69]
Breastmilk	SALLE	UHPLC-MS/MS	N/A	0.66–0.86 ^d^	2.19–2.87 ^d^	[85]
Milk	Extraction with MTBE solvent	LC-MS/MS	90.6–101.2 for PFOA and 89.2–98.4 for PFOS	0.038 for PFOA and 0.002 for PFOS	0.125 for PFOA and 0.007 for PFOS	[74]
Milk, cottage cheese, natural yoghurt, kefir (bonny clabber), sour cream, Camembert-type cheese, and butter	QuEChERS method	Micro-HPLC-MS/MS)	70–120	0.003–0.009	0.010–0.027	[77]
Retail milk and yogurt	UE-MeOH followed by SPE purification with HLB cartridge	HPLC-MS/MS	85.4–90.1 for PFOA and 80.3–84.3 for PFOS	5–10 ^d^ for PFOA and PFOS	15–30 ^d^ for PFOA and PFOS	[78]
Cow milk	QuEChERS method	LC-MS/MS	78.5–111 for PFCAs and 72.8–105 for PFSAs	0.0030 and 0.010 ^d^ PFCAs and PFSAs	0.010 and 0.050 ^d^ PFCAs and PFSAs	[82]

#### Overall Summary

As with meat samples, there are several extraction and analytical techniques for PFAS determination in milk and dairy products. Therefore, a lack in standardization of approaches is still present. So far, the results suggest the high applicability of the QuEChERS method and micro-HPLC-MS/MS for PFAS determination in such matrices. Basically, this is the approach that detected all PFASs analyzed with the lowest RDSs (<10%) and satisfactory recoveries (e.g., 70–120%).

### 4.3. Fruit and Vegetables

The occurrence of PFASs is also strongly investigated in fruit and vegetables. Such matrices may efficiently adsorb PFASs from soil or water contaminated by using biosolids in agricultural practices but also via atmospheric deposition of chemicals on plants [68,86,87]. Fortunately, compared to the other matrices discussed above, foods of plant origin are a minor source of exposure to PFASs. However, a robust and sensitive method of extraction is needed to identify and quantify their low levels. Several studies available in the literature have reported various extraction, cleanup, and analysis procedures. Meng et al. [28] developed an extraction and cleanup method to quantify 45 PFAS (including 3 perfluoroalkyl ether acids (PFEAs), 3 perfluoroalkane sulfonamides (PFASAs), and 6 fluorotelomer carboxylic acids (6:2 FTCA)) in 10 types of fruit and vegetable. Samples spiked with isotopically labelled standard were extracted with 4 mL of basic MeOH, followed by vortexing (30 s), sonicating (30 min), and centrifuging (4000 rpm for 10 min). Matrix cleanup has been accomplished with Oasis WAX SPE cartridges using an automated SPE system designed for PFAS analysis. The MLQs and good recoveries of 38–44 PFASs (Table 3), suggested the capability of the method to identify a wider range of PFASs in food, particularly PFCA, PFSA, and fluorotelomer sulfonate (FTS), most likely present in fruits and vegetables. The extraction procedure by means of ACN followed by Oasis SPE-WAX cleanup validated by Rawn et al. [40] is described in Section 4.1. In particular, they carried out a proficiency test analysis of PFASs on vegetables (lettuce) and fruits (tomato) to evaluate the feasibility of method originally developed in four food matrices (fish, pizza, chicken nuggets, and spinach). The assessment of 12 PFAAs in fruit, cereals, sweets, and salt was performed by D’Hollander et al. [86] using 10 mL of 10 mM KOH/MeOH solution for the extraction. As with previous studies, the resulting solution was undergone to cleanup loading on Oasis WAX cartridges then eluted with 2 mL of 1% NH_4_OH in ACN. Detection rates in the fruit samples showed the highest concentration for PFOS (539 pg g^−1^) followed by PFNA, PFHxS, PFHpA, PFHxA, and PFOA with maximum concentrations of approximately 200 pg g^−1^. Bao et al. [88] analyzed the persistence of PFASs in groundwater and home-produced vegetables and eggs derived from Fuxin fluorochemical industrial park (China) by HPLC-MS/MS. The vegetable and egg samples were twice extracted with 5 mL of MTBE, followed by cleanup with Waters Oasis WAX cartridges. After PFAS elution with 5 mL of acetate (Ac) buffer solution and 10 mL of MeOH, additional cleanup of the extracts was performed with an ENVI-Carb cartridge. Based on the analytical results, PFBA was the dominant contaminant, contributing 67–89% of the total PFASs, followed by PFBuS, which contributed 13–26% of all the target analytes. A similar extraction procedure with MTBE was applied by Sungur et al. [74] to evaluate the presence of PFOA and PFOS in selected foods and beverages (fish, meat, offal, egg, cracker, chip, cake, chocolate, vegetable, milk, and juice). Differently from the previous study, a cleanup of extracts was not performed; the extracts were filtered using a nylon mesh filter (0.2 μM) before LC-MS/MS analysis. The application of a QuEChERS procedure to extract PFASs in fruits and vegetables is also reported by several authors. Genualdi et al. [72] developed a QuEChERS method for the extraction of 16 PFASs in different food products that included 39 fruits and vegetables. The optimizing protocol, described above (Section 4.1), included the investigation of both QuEChERS sorbents (PSA and GCB), the matrix effects, and the SPE cartridges (WAX SPE and Push-Thru WAX SPE). Sznajder-Katarzyńska et al. [87] applied the QuEChERS method starting from a protocol optimized by Surma et al. [89] to evaluate PFCA and PFSA levels in raw fruits and vegetables. The cleanup procedure involved a SOE with 10 mL of ACN and 150 μM of FA with the addition of 4 g of MgSO_4_ and 1 g of NaCl to sample extracts, followed by d-SPE with 0.15 g ENV SPE Bulk Sorbent and 0.90 g MgSO_4_ into a PP 15 mL centrifuge tube. Among PFCAs, PFBA and PFOA were identified in fruit samples with concentrations in the range 0.340–13.450 ng g^−1^ ww for orange and banana, and 0.049–0.448 ng g^−1^ ww for apple and cherry. Within PFSAs, only PFOS was identified in apple samples (0.014 ng g^−1^ ww), whereas two of ten PFASs were detected in vegetable samples. The range of PFOA and PFOS concentrations were 0.049–0.501 ng g^−1^ ww for carrot and tomato and 0.017–2.141 ng g^−1^ ww for white cabbage. Scordo et al. [90] optimized a QuEChERS method to analyze nine PFAAs in strawberry (cultivar “Camarosa”) and olive fruits (cultivar “Frantoio”) by means of LC-MS/MS. Both of samples were extracted with 10 mL of can, with a slight exception for olive samples. Due to their high fat content, the authors applied an ultrasound-assisted procedure (USAE-QuEChERS) consisting of the sonication of the sample for 90 s followed by d-SPE cleanup (400 mg of GCB and 150 mg of MgSO_4_ per mL of extract). Results showed very good linearity for the two methods (R^2^ ≥ 0.9984). However, the use of the USAE-QuEChERS and d-SPE treatment adopted for olive fruit extract, were able to reduce the matrix effect |ME%| to values below 10% for almost all analytes, obtaining higher recoveries (75–95%) compared to strawberry samples (65–89%). The author applied the method to real samples: PFHxS showed a concentration of 148 and 790 pg g^−1^ dw in strawberry (“Market 1” and “Market 2”); PFOA and PFOS were detected only in the “Market 2” sample, at 27 and 90 pg g^−1^ dw. Differently from strawberry, higher concentrations of PFPeA and perfluorobutane sulfonic acid (PFBuS) in both commercial olive fruit were observed. Particularly, all the investigated PFAAs above the MQLs with PFOA and PFOS at 124–137 and 39–95 pg g^−1^ dw were detected in the “Market 2” sample. Zhou et al. [68] developed a modified one-step QuEChERS approach to simultaneously identify 20 PFASs from five representative varieties of vegetable cultivated in a greenhouse (cucumber, lettuce, eggplant, tomato, and leek). To determinate the best conditions for PFAS extraction, various ACN-FA mixtures (0%, 1%, 2%, or 3% v:v FA) were tested. Moreover, two Sin-QuEChERS cartridges were used for the cleanup process. Results reported in the paper showed higher recoveries for solution containing 1% FA for most of the PFASs than for those using 2% or 3% FA. However, taking the purifying effect into account, the authors selected column A (90 mg of PSA and 15 mg of multi-walled carbon nanotubes (MWCNTs)) for simple matrices (tomato, lettuce, and eggplant) and column B (90 mg of PSA, 80 mg of C_18_, 60 mg of GCB, and 15 mg of MWCNTs) for complicated matrices (cucumber and leek). The final validated method tested on real samples showed PFOA as the most frequently occurring compound (0.023–0.153 μg kg^−1^) followed by PFBA and PFPeA detected in some foods with concentrations ranging from 0.025 to 0.371 μg kg^−1^ and from 0.028 to 0.181 μg kg^−1^, respectively. A new QuEChERS method was optimized by Genualdi et al. [91] to evaluate the uptake of PFASs to cranberry fruits and their concentration in water used for the irrigation. The extraction of PFOA and PFOS in cranberry samples was carried out by the addition of 10 mL of ACN and 150 μL of FA to the mixture followed by d-SPE cleanup (0.9 g MgSO_4_, 0.3 g PSA, 0.15 g GCB) of the supernatant before LC-MS/MS analysis. Water bog samples analyzed using the EPA 537 method showed PFOS concentrations ranging from 15 to 122 ng L^−1^ but no PFOA or PFOS was detected in cranberry samples above their MDLs (0.4 and 0.5 ng g^−1^).

#### Overall Summary

The QuEChERS method emerged as the most applied extraction technique for PFAS isolation from vegetables and fruits subsequently analyzed by means of LC-MS/MS, achieving high recoveries (i.e., >75% on average). However, a lack of standardization in approaches has emerged, which could make the comparison of results challenging.

### 4.4. Eggs

Another source of PFAS exposure is eggs and egg products. Their large accumulation, especially LC PFASs, is due to the strong binding affinity towards egg (lipo)proteins contained in high concentrations in yolk [75]. The occurrence of PFCAs, PFSAs, and perfluoroalkyl ether carboxylic acids (PFECAs) by means of different extraction and detection methodologies was investigated by several authors (Table 4). Sungur et al. [74] detected the highest PFOS concentration in egg samples (4.587 ng g^−1^) in relation to other food items analyzed (fish, meat, offal, cracker, chips, cake, chocolate, vegetable, milk, and juice). Extraction was carried out by the addition of 5 mL of MTBE to samples previously spiked with 1 mL of 0.5 M tetrabutylammonium hydrogen sulphate solution (Bu_4_NHSO_4_) and 2 mL of (Na_2_CO_3_) buffer (0.25 M, pH = 10). Lasters et al. [92] examined the prevalence of 17 PFASs in home-produced eggs collected in a different area located within in a 10 km radius of a fluorochemical plant site in Antwerp (Belgium). Samples spiked with ISTD solution were extracted by the addition of 10 mL of ACN. For the cleanup procedure, three types of sorbents were tested: graphitized Envi-Carb powder, WAX-SPE, and a combination of them. Because of the high recovery capacity, the graphitized Envi-Carb cartridge was finally selected. On the basis of the results obtained, PFOS and PFOA were the most frequently occurring compounds detected in all egg samples. The range of PFOS concentration was 0.13–241 ng g^−1^ ww and decreased with increasing distance from the fluorochemical plant site. Moreover, the concentrations of PFOA were higher in young laying hens than in relatively old laying hens (*p* = 0.07 and *p* < 0.01, respectively). The frequent detection of PFASs in chicken eggs was explained by the feeding of free-range laying hens from contaminated soil particles, invertebrates in contact with the soil, and kitchen leftovers, which is different from commercially produced chickens kept indoors and fed with commercial feed [93]. The influence of the laying hens’ age on PFAS concentration could be explained by maternal transfer and lower elimination capacities at around 16 months of age onwards compared to other birds. The analytical method developed by Berendsen et al. [75] for PFAS analysis in milk (Section 4.2) was also applied on two egg samples collected from two high-risk PFAS exposure areas in the Netherlands. Results suggested that the addition of a saturated Pb (OAc)_2_ solution during alkaline extraction promoted efficient protein precipitation and clearer extracts after the purification through Strata-X-AW SPE cartridges. Moreover, only one of the two eggs showed a PFOA level, detected at 0.14 ng g^−1^. Tahziz et al. [93] validated an analytical method for PFOS and PFOA determination in the yolk of poultry eggs in Malesia. They attempted several combinations of cleanup and extraction procedures (alkaline digestion, simple protein precipitation, UE, and SPE). All techniques evaluated showed low recoveries (<60%) except the simple protein precipitation technique, which showed greater recoveries (range: 84–102%) and was consequently chosen for the analysis of 47 poultry egg yolk samples. ACN was the extraction solvent selected to promote the aggregation and precipitation of proteins, and 3 mL was added to the egg yolk. After centrifugation and the removal of the protein pellet, the supernatant was dried, dissolved in 1 mL of MeOH, vortexed, and filtered through a 0.2 μm syringe filter. Results reported from LC-MS/MS analysis showed that six egg yolk samples (five home-produced chicken eggs and one quail egg) contained PFOS concentrations ranging between 0.5 and 1.01 ng g^−1^. The PFOA concentration was not detected at quantifiable levels (<0.1 ng g^−1^). In addition, unquantifiable concentrations of PFOS (<0.50 ng g^−1^) and PFOA (<0.10 ng g^−1^) in commercially produced eggs confirmed the lower exposure of commercially produced chickens to PFASs due to their feeding with high-grade natural feed (grains, herbs, and fruits). Zafeiraki et al. [94] investigated PFAS contamination in home-produced eggs from the Netherlands and Greece, comparing it to commercially produced eggs. They extracted a sample spiked natively and labelled PFASs by means of alkaline digestion. The solution was added with 10 mL of MeOH, vortexed for 1 min, and shaken for 30 min at 250 rpm. To neutralize the solution and to remove insoluble particles, 150 μL HCl was added to MeOH extract. The supernatant recovered after centrifugation (10 min at 10,000 rpm) was cleaned through Oasis WAX SPE cartridges, eluted with 2 mL of 2% NH_4_OH in ACN, dried, and dissolved in 775 μL of 2 mM AF in water and 200 μL of MeOH before LC-MS/MS analysis. Levels of PFASs detected were higher in home-produced eggs from the Netherlands (median 3.5, range < LOQ-31.2 ng g^−1^) and Greece (median 1.1, range < LOQ-15.0 ng g^−1^) than in eggs collected from supermarkets. Differently to home-produced eggs, commercial egg samples showed all PFASs below the LOQ of 0.5 ng g^−1^, except for PFOS, found at 1.1 ng g^−1^ and 0.9 ng g^−1^ in one organic egg from the Netherlands and one free-range egg from Greece, respectively. PFOS was the predominant compound revealed, with the highest concentration in a Dutch home-produced egg sample (24.8 ng g^−1^). Other LC PFASs (PFOA, PFNA, PFDA, and PFUnDA) were detected in the range between 2% and 36% of the samples. As in the previous study, the greater level of PFASs in home-produced eggs can be attributed to ingestion of contaminated soil particles and small organisms by free-range home-kept laying hens. Significant levels of PFASs were monitored by Gazzotti et al. [95] in eggs from backyard chickens (EBCs) raised in Italy. The yolk samples were extracted using alkaline digestion followed by SPE. The extraction procedure performed was based on a protocol reported by Zaferaki et al. [94]. PFOS was the most abundant compound detected with the highest concentration (up to 3.47 μg kg^−1^ and a median value of 1.29 μg kg^−1^) among the quantifiable samples. This value was in line with the values reported for eggs collected in Malesia (1.01 ng g^−1^) and Greece (1.1 ng g^−1^) and lower than that obtained for eggs collected in the Netherlands (3.5 ng g^−1^). However, high contaminant levels were reported in EBCs collected in northern Italy compared to EBCs from the central/southern region. This condition could be related to the several industrial plants placed mainly in northern Italy. The analytical method developed and validated by Sadia et al. [69] was also applied to identify PFOS and PFOA in egg matrices. In order to reduce the presence of taurodeoxycholic acid (TDC), an interfering compound eluted at the same retention time as PFOS, an additional cleanup with ENVI-Carb SPE (100 mg, 250 mg) tube was performed. The final analysis of three egg samples showed that increasing the amount of ENVI-Carb enhanced the efficiency of eliminating TDC. As matter of fact, TDC was detected only in the first and second egg samples (without and with 100 mg ENVI-Carb) but not in the third sample, which was purified with 250 mg ENVI-Carb. Moreover, the method applied on real egg samples detected the highest PFOA and PFOS concentration in chicken eggs (4.3 pg g^−1^ and 211 pg g^−1^, respectively) but it was not validated for PFHxS determination due to the interfering substances co-eluted with the latter. The QuEChERSER method validated by Taylor and Sapozhnikova [67] for beef, chicken, catfish, and pork was also applied for the analysis of PFASs in liquid and powdered eggs. Comparing the QuEChERSER, FDA, and FSIS methods, the authors suggested that all three methods showed similar recoveries in eggs ranging between 70 and 120%. In addition, the automated instrument top sample preparation (ITSP) evaluated for cleanup of QuEChERSER extracts provided an efficient alternative to the traditional d-SPE to minimize ME (±20%). Finally, the application of HRMS allowed for differentiating PFOS with cholic acid (CA) interferents in eggs, decreasing the chances of a false positive.

#### Overall Summary

The best methodologies that could be suggested for extraction and analysis of PFASs are the alkaline digestion SLE with ACN as extraction solvent, followed by SPE-WAX cleanup or QuEChERSER extraction. The analyses carried out with LC-MS/MS and UHPLC-MS/MS QqQ, respectively, showed satisfactory recoveries and LODs (i.e., 76–93%, 89 ± 9%, 0.0011–0.0016, and 0.0003 ng g^−1^, respectively).

### 4.5. Fish and Shellfish

Detectable concentrations of PFASs have been observed in fish and shellfish globally. These aquatic organisms can assimilate perfluoroalkyl substances from water, suspended solids, and sediment through the gills and filtered feedings [40,96]. Liver is the organ where PFASs mainly accumulate due to its high affinity for hepatic proteins. However, kidneys also show a higher PFAS concentration than muscle and other fish tissues [97]. Several analytical methods have been developed to extract and to identify PFASs in seafood (Table 5). Ciccotelli et al. [98] validated a rapid analytical method to assess PFOA and PFOS in freshwater fish (European perch) from Lake Maggiore and in cereal from Piedmont study area (Biella district). To achieve the best extraction procedure, they tested different techniques (namely, A–E). Due to the better recoveries of PFOA and PFOS (51% and 110%, respectively), their repeatability (18% and 15%, respectively), and to the simple and rapid execution, procedure A was selected for fish muscle extraction. Samples fortified with 50 uL of IS (C_8_PFOA, 100 ng mL^−1^) were mixed with NaOH (2.5 mL) and extracted with 10 mL of MeOH. The next purification of the extracts was performed by means of Water Oasis WAX cartridges. The LC-MS/MS analysis of real samples showed a detectable concentration of PFOS in all fish samples (Coregonis lavaretus and Perca fluviatilis), ranging from 5 to 45.80 ng g^−1^, whereas low PFOA levels below the LOQ were found. Due to the high intake of marine shellfish in South Africa, Abafe et al. [99] investigated the occurrence of PFASs and their potential human health risk. They extracted the homogenized fish samples spiked with 0.2 ng g^−1^ of a mixture of C_3_-PFOA and C_3_-PFNA with 10 mL each of ACN and water. After the salt addition (4 g of MgSO_4_ and 1 g of NaCl) and centrifugation (800 rpm for 10 min), a UHPLC-MS/MS analysis was carried out for chromatographic separation of PFASs. Results reported using the validated method showed different recoveries of PFASs in ranges between 57 and 109% (abalone), 63 and 119% (lobster), 82 and 146% (mussel), and 63 and 124 (oyster) for the single fish sample analyzed. PFPeA, PFOS, PFHxA, and PFTeDA were the most prevalent compounds detected in the range frequencies at 94, 88, 76, and 71%, respectively. The highest concentrations were found in mussels (range 4.83–6.43 ng g^−1^), which are able to accumulate organic contaminants within their sessile and filter-feeding organism. The occurrence of PFOS and PFOA concentrations in seafood was also carried out by Lertassavakorn et al. [100]. Seafood samples spiked with isotopic-labelled ISTD (C_8_-PFOS and C_8_-PFOA) were extracted with 10 mL of MeOH. After, the extracts were shaken in a water bath (30 °C for 90 min), centrifugated (4000 rpm for 5 min), and cleaned through Oasis WAX-SPE cartridges. The UHPLC-MS/MS analysis reported higher PFOS concentrations than the LOQ level (Table 5) and PFOA. More precisely, the highest concentration of PFOS was found in squid (6724 ng kg^−1^ ww) whereas the highest concentration of PFOA (421 ng kg^−1^ ww) was found in cockle samples. Monitoring of 20 PFASs in a fish species (Alosa agone) of Italian subalpine lakes was conducted by Mazzoni et al. [101]. Solvent extraction (SE) by means of sonification in an acidified water and ACN 10:90 *v*/*v* solution was applied for spiked fish samples. The extracts were purified on MgSO_4_/NaCl, filtered through Hybrid-SPE^®^-Phospholipid Ultra-cartridges, were analyzed by UHPLC-MS/MS coupled to turbulent flow chromatography (TFC). All samples tested showed PFOS levels ranging between 0.4 and 16.6 ng g^−1^. Differently from the fish of the other subalpine lakes where PFOS concentration was lower (3.1 ± 1.9 ng g^−1^ ww), Lake Maggiore showed significantly higher levels of PFOS (mean = 13.0 ± 3.2 ng g^−1^ ww), which exceeded the European EQSbiota (9.1 ng g^−1^ ww). Only one sample in Lake Maggiore showed a relevant level of PFOA (1.2 ng g^−1^ ww). On the other hand, no large difference in LC chain PFCA concentration among the lakes (0.3 to 2.7 ng g^−1^ ww) was found. An overview of the concentration of PFASs in the liver, fillet, and belly flap of beaked redfish and cod was conducted by Kowalczyk et al. [102]. They twice extracted the samples with 10 mL of MeOH and, after combining, all supernatants underwent centrifugation (5 min at 3500 rpm), purification, and concentration by Oasis WAX-SPE column. PFOA and PFUnDA were the most frequent substances quantified in the liver and fillet of beaked redfish and cod, and in the belly flap of beaked redfish in a range between 40 and 100%. Chen et al. [97] investigated the accumulation levels of PFAAs, perfluorooctane sulfonic acid precursors (PreFOSs), and PFASs and in different types of fish sampled from Taihu Lake (Meiliang area). Fish samples were twice extracted with 5 mL of MTBE solution, shaken (20 min at 250 rpm), and centrifuged (600 rpm for 10 min). The final extracts were combined and purified through GBC-SPE cartridge. Seven PFAAs (PFOA, PFNA, PFDA, PFUnDA, PFDoDA, PFHxS, and PFOS) and one PreFOS, namely, perfluorooctane sulfonamide (PFOSA), were detected in all fish tissue samples. PFOS was predominantly observed (range: 0.349–331 ng g^−1^ ww), followed by PFUnDA and PFDA (range: 0.0900–76.0 ng g^−1^ ww and 0.207–73.4 ng g^−1^ ww, respectively). On the other hand, the high concentration of PFOSA (0.00108–5.82 ng g^−1^) ww found can be explained by the presence of a major transformation intermediate in many PreFOSs. In addition, the main PFAS concentrations were observed in livers and kidneys (range: 107–481 ng g^−1^ ww and 10.6–530 ng g^−1^ ww, respectively), whereas the lowest levels of PFASs were observed in the muscles (1.26–34.6 ng g^−1^ ww). The occurrence of POPs was studied by Courderc et al. [103]. They evaluated the levels and profiles of several compounds (polychlorinated biphenyls (PCBs), polybrominated diphenyl ethers (PBDEs), alkylphenols (APs), polycyclic aromatic hydrocarbon metabolites (OH-PASs), bisphenol A (BPA), and PFASs in the muscles and bile of European eel (Anguilla Anguilla) using it as chemical sentinel species for level contamination monitoring of the Loire estuary (France). The analysis of PFASs was carried out as follows: 1 g of freeze-dried eel muscle spiked with 2 ng of ISTD mixture (10 13C mass-labeled compounds) was extracted by SLE using 15 mL of 0.01 M KOH of MeOH. After concentration under a nitrogen stream, the supernatant was recovered and twice purified: firstly, using a WAX-SPE cartridge, and secondly, using Envi-Carb stationary phase. Measurements performed by means of LC-MS/MS showed the sum of PFASs in the range from 75.25 to 123.67 ng g^−1^ dw. PFOS and PFUnDA were detected in the muscles of all eels whereas SC PFCAs (PFBA, PFPeA, and PFHpA) and PFOA were not found. Moreover, levels of PFOS were predominant in yellow eels (range: 67.6–106.9 ng g^−1^ dw) whereas the level for silver eels was 61.6 ng g^−1^ dw. The higher detection could be explained by the industrial activities (paper and metal surface treatment) that represent potential sources of PFOS around the Loire estuary. Analytical methods developed by Rawn et al. [40] and Sungur et al. [74] were also applied to fish. The results of the studies showed a low mean recovery for PFHxA, FOUEA, PFDoA, and perfluododecane sulfonate (L-PFDoS) ranging between 49.1 and 63.0% in canned fish, high recoveries of 6:2 PAP and 8:2 PAP (169 and 135%, respectively), and a prevalent content of PFOA and PFOS (range: 0.147–0.543 ng g^−1^ and 7.96–52.43 ng g^−1^, respectively) in carp, conger, pikeperch, horse mackerel, sardine, and black cod samples. Several authors applied the QuEChERS method to identify the levels of PFASs in fish [67,70,72,96]. They carried out a first extraction with 10 mL of ACN followed by d-SPE cleanup of fish samples with different sorbents (MgSO_4_, PSA, C_18_, CGB, ENVI-Carb, and WAX) tested. Acceptable recoveries were showed in catfish (84%), fish muscle (77.7–120.2%), tuna (44–116%), salmon (52–120%), and shrimp (72–120%) [67,96]. In addition, levels of PFOS were detected in two tilapia samples (83 and 87 ng kg^−1^) [72] and crabs (0.9–4.6 μg kg^−1^), a high level of PFOA (3–20 μg kg^−1^) in clams, PFUnDA and PFDoA in cod (55–332 ng kg^−1^) and tuna (36–888 ng kg^−1^), a low level of PFDoA in salmon (less than 0–045 μg kg^−1^) and shrimps (0.027 μg kg^−1^), and only LC PFCAs in pollock (28–284 ng kg^−1^) and trout (27–4215 ng kg^−1^) [70,96]. Finally, several papers published in the literature used digestion techniques for PFAS determination in fish. For example, Ruffle et al. [104] analyzed the type and concentration of PFAS in finfish/shellfish samples of U.S. origin by means of digestion and SPE. Results of UPLC/MS/MS analysis showed that the concentration of PFASs was higher in freshwater fish from the Midwest region (yellow perch fillet, whitefish, and walleye) with the predominant levels of PFOS (range: 1.2–19.1 ng g^−1^). Analytical methods developed by Sadia et al. [69], explained in Section 4.1, Section 4.2 and Section 4.4, were also applied to fish. They obtained good recoveries (75–79%) with a detectable concentration of PFASs (1.2–250 pg g^−1^) in real fish samples classifying PFOS and PFOA as the major compounds found at concentration levels of 4.15 pg g^−1^ and 55.3 pg g^−1^, respectively. Taylor et al. [105] studied a novel experimental design to test depuration of PFASs from edible tissues of portunid crabs (Giant Mud Crab Scylla serrata). The extraction procedure performed was based on the USEPA 537 method described by Taylor et al. [106]. Briefly, 1 g of crab tissues were digested with 0.4 mL 200 mM MeOH and extracted with 4 mL of ACN. The resulting extracts were firstly purified by means of LLE with n-hexane (C_6_H_14_) followed by cleanup through Bond Elut carbon cartridges. HPLC-MS/MS analysis showed a significant decrease in PFOS, PFHxS, and PFOA concentration with depuration half-lives up to 40 days for PFOS. However, a variability in PFAS concentration was also revealed between claws from the same individuals. This was potentially due to claw loss and regrowth prior to capture.

#### Overall Summary

PFAS extraction from fish with MTBE solvent followed by cleanup with GBG-SPE cartridge coupled with the UPLC-MS/MS provided recovery ranging 53–116% with LOD ranging 0.0002–0.056 ng g^−1^. However, approaches are still lacking in harmonization and standardization, leading to a challenging comparison of the results.

### 4.6. Honey

Honey is a natural foodstuff highly consumed by children, the elderly, and pregnant women.

It contains approximately 300 different compounds of which the majority are sugars in water (80–83%). The remaining substances are proteins, lipids, vitamins, enzymes, phenolic acids, volatile chemicals, flavonoids, organic acid, amino acids, and minerals [89,107]. Due to its health and nutritional properties, it is necessary to control its quality and safety, which could be potentially compromised by the presence of a various contaminants such as polycyclic aromatic hydrocarbons (PAHs), heavy metals, plasticizer residues, and PFASs in the environment [108]. Based on scientific researchers’ focus on the assessment of PFASs in food, only two papers by Surma et al. were found for honey in the literature [83,109].

**Figure 1:**
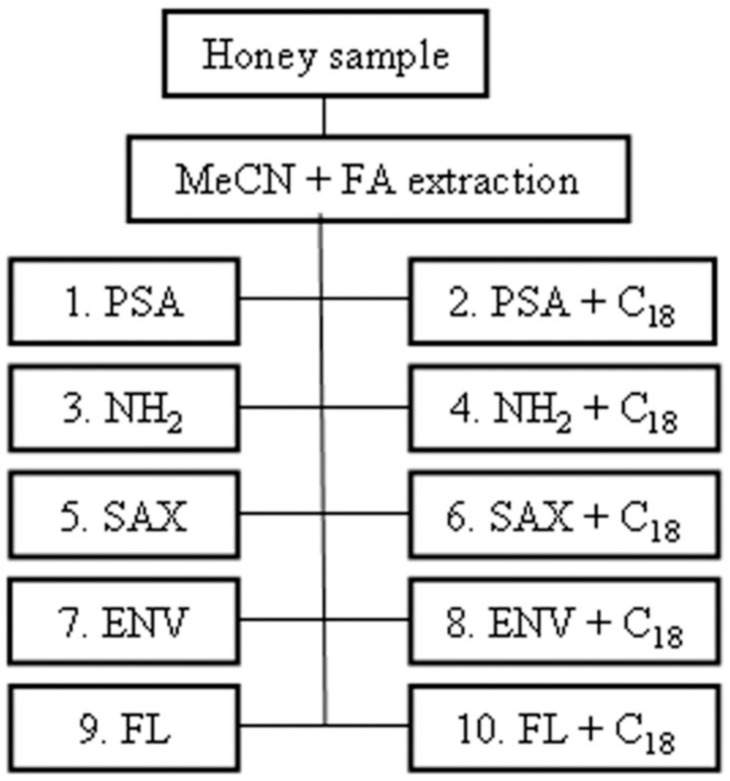
Scheme of sorbents tested in the study.

**Figure 2:**
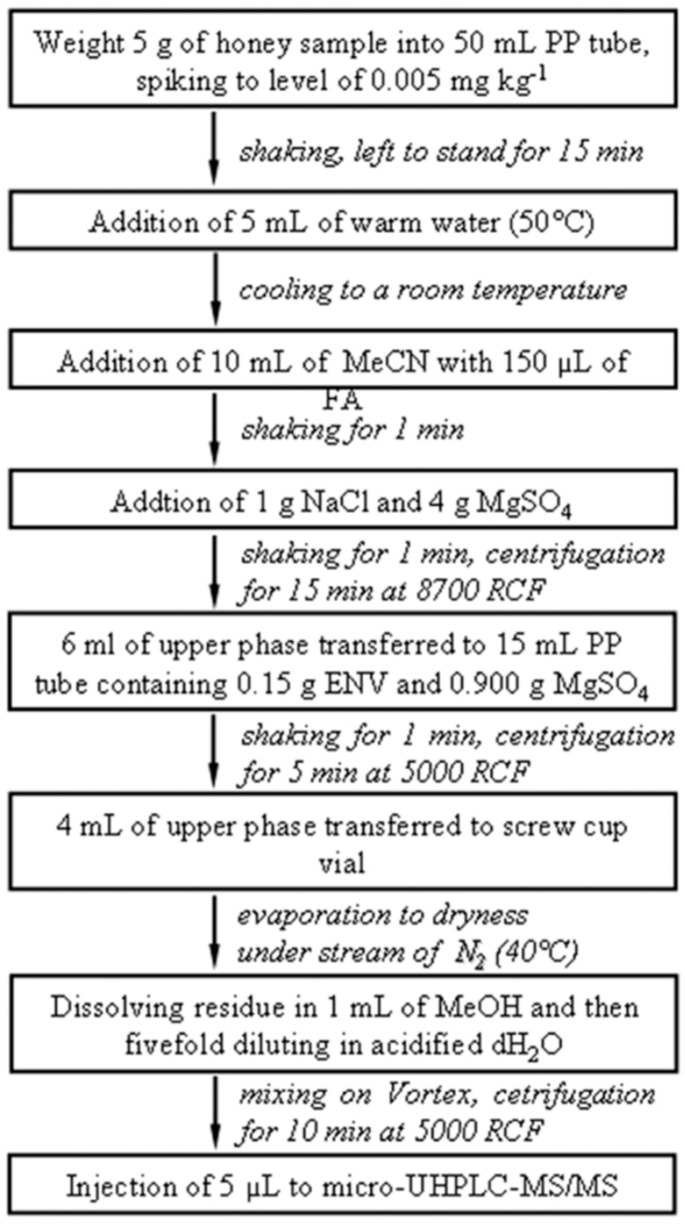
Scheme of the final analytical procedure applied.

In the first paper, published in 2015, [83] the authors developed an analytical method based on (d-SPE) and micro-UHPLC-MS/MS analysis to identify the presence of PFOA and PFOS in honey samples. A series of experiments based on the selection of appropriate stationary phase were conducted to optimize the sample preparation step (Figure 1). However, the final extraction procedure applied was as follows: 5 g of honey spiked with ISTD mixture was extracted using 10 mL of ACN and 150 μL FA. After the addition of 1 g NaCl and 4 g MgSO_4_, the supernatant was transferred into a PP 15 mL tube containing selected sorbents (0.15 g ENV and 0.900 g MgSO_4_). The extracts obtained underwent evaporation under a stream of N_2_, recovered with 1 mL of MeOH, and analyzed by UHPLC-MS/MS (Figure 2). The selection of method 7 was based on the recoveries of PFOA and PFOS (Table 6) being better than for the other sorbents tested (PSA, SAX, NH_2_, FL, and C_18_), which showed recovery ratios in the range 40 to 84% for PFOA and 47 to 87% for PFOS. The application on real samples showed PFOA levels in the range of 0.10–0.223 ng g^−1^ for French acacia honey and Spanish heather honey, respectively. Only two honey samples (Italian eucalyptus and Spanish heather) showed PFOS levels below LOQ. According to the method validated above, the second paper, published in 2016 by Surma et al. [109], investigated the levels of 10 PFASs in honey samples selected from eastern, northern, and southern European countries. PFCAs were detected in almost all honey samples (range: 0.124–0.798 ng g^−1^), PFHpA was mostly detected in Polish and Slovak honey samples (mean: 0.309 and 0.191 ng g^−1^, respectively), whereas PFOA and PFNA were mainly detected in honey from Spain (0.071 ng g^−1^ for thyme, 0.253 ng g^−1^ ww for heather). The highest concentration and variety of PFASs (0.878 ng g^−1^ ww) was detected in Italian eucalyptus honey, in which only PFDA was quantified (0.278 ng g^−1^ ww). The difference in PFAS distribution observed between two Polish regions was explained by the industrialization of the Malopolska region compared to Warmia and Marzury, which were recognized as “the green lungs of Poland”. On the contrary, the different climates, environments, and socioeconomic conditions of the three geographic European regions did not influence the PFAS contamination levels of the honey samples collected.

### 4.7. Beverages

Drinking water contamination is mainly due to the failure of drinking water treatment plants to remove PFASs [110]. Table 7 summarizes the main analytical methods used for the determination of PFASs in aqueous matrices. For example, Olomukoro et al. [110] evaluated a SPME-LC-MS/MS method for the determination of PFOA, PFOS, and PFBuS in water samples using hydrophilic–lipophilic balance–weak anion exchange/polyacrylonitrile (HLB-WAX/PAN) sorbent. The LOQs and method linearity (1–5000 ng L^−1^) achieved by the protocol exceeded EPA regulatory limits for PFASs in drinking water, establishing SPME as a reliable preconcentration method for the ultra-trace analysis of PFASs. The cleaning and sample handling processes can affect the recoveries of these analytes and can lead to contamination during the analytical process. On the other hand, the use of the DI approach can lead to matrix effects. However, DI-LC-MS/MS can be applied to drinking water, where the matrix effect is less important than in wastewater, surface water, or groundwater. According to the study just discussed [110], Ciofi et al. [111] determined concentrations of PFOA and PFOS (12.8 ng L^−1^ and <0.16 ng L^−1^, respectively) as being lower than the limits recommended by the Italian Health Institute (ISS) in drinking water samples (0.5 µg L^−1^ and 0.03 µg L^−1^, respectively). In addition, a new sample pretreatment technique is Magnetic SPE (MSPE). This method consists of the separation of the target analytes by means of magnetic adsorbents dispersed in the sample solution. Xian et al. [112] applied a novel fluorine- and nitrogen-functionalized magnetic graphene (G-NH-FBC/Fe_2_O_3_) material as an adsorbent in MSPE to extract PFASs in water and functional beverages. The following detection was carried out by the use of an HPLC-Orbitrap HRMS system. Results of the study confirmed the accuracy of the MSPE method and HPLC-Orbitrap HRMS combined with good recoveries and a wide linearity range. On the other hand, this method is not very sensitive or suitable for samples containing ultra-trace contaminants. Besides water, the presence of PFASs has also been investigated in other beverages such as juices and tea. For example, Igarashi et al. [113] monitored the presence of PFASs in bottled water, tea, and juice samples using an SPE cartridge (Presep PFC-II) for the cleanup and concentration of samples followed by quantification by LC-MS/MS. Results obtained by LC-MS/MS analysis showed acceptable recoveries for all three matrices tested. Bubble tea is another drink predominantly consumed worldwide among children and young people. Huang et al. [80] investigated the presence of PFASs in some fast foods, including bubble tea. For the analysis, the bubble tea was sampled without the “pearls” (mainly made up of tapioca). After a UE with ACN solvent, the samples were cleaned and concentrated through an OASIS WAX SPE cartridge, and subsequently analyzed by UHPLC-HRMS. Isotope-labelled surrogates were used to determine recoveries. The technique applied allowed for achieving good recoveries, except for MFHEA, in which not very satisfactory recoveries were observed (43–72%). This could be due to its instability in the mass spectrometer and the influence of complicated matrices.

#### Overall Summary

Analyses of beverages were performed using several approaches, confirming a lack in the selection of unique analytical methodologies. The literature has suggested several satisfactory methods; however, ASE and SPE cleanup with an Oasis WAX cartridge coupled with UPLC-MS/MS provided recovery ranging 80.2–95.2% with the lowest LOD (i.e., 0.00008–0.00068 ng g^−1^).

## 5. Conclusions

The emerging presence of PFASs in the environment has become a serious issue because of their resistance to hydrolysis, photolysis, and biodegradation processes.

The ingestion of contaminated foods is the main pathway of human exposure to PFASs, which could be deleterious for their toxicological effects on health.

Several extraction procedures based on LLE, SPE, or alkaline digestion were investigated for the separation of PFASs from foods. In addition, a lot of SPE sorbents (Oasis WAX, Water Oasis HLB, ENVI-Carb, Strata X) were tested to avoid possible artefacts connected with the complexity of the matrix tested and to purify the sample prior to or after the extraction phase. Another goal for the assessment of PFASs concerned sensitive detection through chromatographic techniques such as LC-MS/MS, HPLC-MS/MS, UHPLC-MS/MS, UPLC-MS/MS, and GC-MS using the ESI-MS/MS, QqQ, and HRMS systems to reach high recoveries and low LODs and LOQs. Recently, the development of the QuEChERS method resulted an efficient, fast, and economic alternative for extraction of PFASs. Moreover, the implementation of optical and electrochemical procedures based on colorimetric and fluorometric essays with colloidal nanoparticles (NPs), redox dyes, molecularly imprinted polymers (MIP), CdS quantum dots (QD), and electrochemical modified sensors based on MIPS, graphitic carbon, polymers, NPs, and metal–organic frameworks (MOFs) could represent an innovative and future trend for the rapid investigations of PFASs. However, based upon the fact that these sensors lack sensitivity and do not satisfy the regulatory detection limit, a synthesis of novel material with specific binding sites for PFASs will be investigated at a later point.

## Figures and Tables

**Table 3:** Summary of analytical procedure for the analysis of PFASs in fruits and vegetables and their recoveries (expressed as%), LODs (ng g^−1^), and LOQs (ng g^−1^). N/A = not available; ^a^ = MQL; ^b^ = MDL.

Matrix	Extraction and Pretreatment	Analysis	Recoveries	LOD	LOQ	Ref.
Home-produced vegetables (tomato, cucumber, eggplant, pepper, Chinese cabbage)	Extraction with MTBE solvent followed by cleanup SPE with Water Oasis WAX cartridge	HPLC-MS/MS	82–95	N/A	0.20	[88]
Berries, citrus fruit, pipe and stone fruit, melons, grape, bananas	Extraction with KOH/MeOH solution followed by SPE cleanup with Oasis WAX cartridge	UPLC-MS/MS	67–106	N/A	0.001–0.04	[86]
Cranberry	QuEChERS method	LC-MS/MS	69–109	0.20–5.6	N/A	[91]
Strawberries, Brussels sprouts pepper, green beans, cabbage, collards, squash, apple, asparagus, avocado, banana, peaches, blueberries, broccoli, cantaloupe, carrot, cauliflower, celery, corn, cucumber, eggplant, lettuce, mushrooms, onion, orange, pear, pineapple, spinach, potato, tomato, watermelon, grapefruit	QuEChERS method	LC-MS/MS	40–120	0.02–0.107	N/A	[72]
Blackberries, blueberries, corn kernels (corn), grapes, okra, peaches, pecans, potatoes, squash, and tomatoes	Extraction with basic MeOH solvent followed by SPE cleanup with Oasis WAX SPE cartridge	LC-QqQ-MS	50–150	N/A	0.025–0.25 ^a^	[28]
Lettuce, spinach, and tomato	Oasis SPE-WAX cleanup and extraction with ACN solvent	LC-MS/MS	47–252	0.006–1.76 (L-FDS and FHEA, respectively)	0.018–5.28 (L-FDS and FHEA, respectively)	[40]
Strawberry and olive fruits	QuEChERS method	LC-MS/MS	65–89 (strawberry) and 75–97 (olive fruits)	0.0007–0.109 ^b^	0.0029–0.393 ^a^ (strawberry) 0.0026–0.127 ^a^ (olive fruits)	[90]
Vegetable (parsley, tomato, potato, peas)	Extraction with MTBE solvent	LC-MS/MS	91–101 (PFOA) and 89–98 (PFOS)	0.038 (PFOA) and 0.002 (PFOS)	0.125 (PFOA) and 0.007 (PFOS)	[74]
Banana, apple, lemon, orange, cherry and strawberry, potato, beetroot, carrot, white cabbage, and tomato	QuEChERS method	Micro-HPLC-MS/MS	85–97	0.002–0.009	0.006–0.024	[87]
Cucumber, lettuce, eggplant, tomato, and leek	QuEChERS method	LC-MS/MS	55–119	0.003–0.034	N/A	[68]

**Table 4:** Summary of analytical procedure for the analysis of PFASs in eggs and egg products and their recoveries (expressed as%), LODs (ng g^−1^), and LOQs (ng g^−1^). N/A = not available; ^a^ = detection frequencies (Freq (%)); ^b^ = lower limit of quantification (LLOQ).

Matrix	Extraction and Pretreatment	Analysis	Recoveries	LOD	LOQ	Ref.
Eggs	Extraction with Pb(OAc)_2_ solution, MeOH, and FA followed by SPE cleanup with Strata-X-AW cartridge	UHPLC-MS/MS	N/A	0.025–2.5	0.025–5	[75]
Eggs and egg products	Alkaline digestion with a NaOH followed by SPE	UPLC-MS/MS	N/A	0.25	0.10	[95]
Home-produced eggs	Extraction with ACN solvent and cleanup using graphitized Envi-Carb powder cartridge	UPLC-MS/MS	12–35 (PFCAs) and 25–51 (PFSAs)	11–100 (PFCASs) ^a^ and 0–100 (PFSAs) ^a^	0.080–0.21 (PFCAs) and 0.13–2.5 (PFSAs)	[92]
Eggs	Alkaline digestion, SLE with ACN solvent followed by SPE-WAX cleanup	LC-MS/MS	76–93	0.0011, 0.0014 and 0.0016 for PFHxS, PFOA, and L-PFOS	0.0031, 0.0034 and 0.0049 for PFHxS, PFOA, and L-PFOS	[69]
Eggs	Extraction with MTBE solvent	LC-MS/MS	91–101 for PFOA and 89–98 for PFOS	0.038 (PFOA) and 0.002 (PFOS)	0.125 (PFOA) and 0.007 (PFOS	[74]
Yolk and poultry eggs	Simple and rapid protein precipitation extraction with ACN solvent	LC-MS/MS	93–102 (PFOS) 84–91 (PFOA)	0.1 (PFOS) and 0.02 (PFOA)	0.5 (PFOS) ^b^ and 0.1 (PFOA) ^b^	[93]
Liquid and powdered eggs	QuEChERSER method	UPLC-HRMS, UHPLC-MS/MS QqQ	89 ± 9	HRMS Method: Liquid eggs (0.0015–0.0161), powdered eggs (0.0003–0.0369) QqQ Method: Liquid eggs (0.003–0.036), powdered eggs (0.005–0.118)	HRMS Method: Liquid eggs (0.0045–0.0489), powdered eggs (0.0009–0.1119) QqQ Method: Liquid eggs (0.008–0.142), powdered eggs (0.016–0.118)	[67]
Home- and commercially produced chicken eggs	Alkaline digestion with NaOH with MeOH as solvent extraction followed by SPE cleanup with Oasis WAX SPE cartridge	LC-MS/MS	60–115	0.15	0.5	[94]

**Table 5:** Summary of analytical procedures for the analysis of PFASs in fish and shellfish and their recoveries (expressed as%), LODs (ng g^−1^), and LOQs (ng g^−1^); N/A = not available; ^a^ = CCα; ^b^ = CCβ; ^c^ = MDL; ^d^ = ng mL^−1^.

Matrix	Extraction and Pretreatment	Analysis	Recoveries	LOD	LOQ	Ref.
Marine Shellfish	Extraction with ACN solvent and water	UHPLC-MS/MS	77–119	0.03–0.1 ^a^ 0.05–0.16 ^b^	0.005–0.05	[99]
Fish	Extraction with MTBE solvent followed by cleanup with GBC-SPE cartridge	UPLC-MS/MS	53–116	0.000200–0.0560 ^c^	N/A	[97]
Fish	Extraction with MeOH solvent followed by SPE cleanup with Water Oasis WAX cartridge	LC-MS/MS	99–102 (PFOA) and 96–108 (PFOS)	0.20–0.47	0.50–0.70	[98]
Muscles and bile of European eel (*Anguilla anguilla*)	Liquid solid extraction (LSE) with KOH of MeOH followed by purification with SPE WAX cartridge and second cleanup with Envi-carb cartridge	LC-MS/MS	47.6–92.4	0.003–0.46 ^d^	0.006–1.259	[103]
Fish	QuEChERS method	LC-MS/MS	67.9–120.2	0.00369–0.017.33	0.05 except for GenX and C_6_O_4_ (0.1 ng g^−1^)	[70]
Salmon, tilapia	QuEChERS method	LC-MS/MS	40–120	0.021–0.09 ^c^	N/A	[72]
Beaked redfish and cod	Extraction with MeOH solvent followed by cleanup with Oasis WAX column	HPLC-MS/MS	90–100	0.2–1.0 (liver), 0.05–0.2 (Belly flap and fillet)	0.3–2.0 (liver), 0.1–0.2 (Belly flap and fillet)	[102]
Seafood	Extraction with MeOH solvent followed by SPE cleanup with Oasis WAX SPE cartridge	UHPLC-MS/MS	94.7–100.8	N/A	0.024–0.048	[100]
Fish	SE in acidified water and ACN solution followed by purification with Hybrid-SPE^®^-Phospholipid Ultra cartridges	UHPLC-MS/MS coupled with TFC	N/A	0.1–1.2	0.03–0.4	[101]
Fish	Oasis SPE-WAX cleanup and extraction with ACN solvent	LC-MS/MS	57.5–116	0.006–1.76	0.018–5.28	[40]
Fish and shellfish	Digestion and SPE	UPLC-MS/MS	N/A	0.412–0.560 and 0.698–0.947 (PFNS)	0.98–1.3	[104]
Fish	Alkaline digestion, SLE with ACN solvent followed by SPE-WAX cleanup	LC-MS/MS	>70	0.0011, 0.0014 and 0.0016 for PFHxS, PFOA and L-PFOS	0.0031, 0.0034 and 0.0049 for PFHxS, PFOA and L-PFOS	[69]
Fish	Extraction with MTBE solvent	LC-MS/MS	89.2–98.4 (PFOS) and 90.6–101.2 (PFOA)	0.002–0.007 (PFOS) and 0.038–0.125 (PFOA)	0.007–0.125	[74]
Crabs	Digestion with NaOH in MeOH and SE with ACN, purification with LLE with C_6_H_14_ solvent followed by cleanup through Bond Elut carbon cartridges	HPLC-MS/MS	N/A	1.2 (PFOS)	N/A	[105]
Catfish tissue	QuEChERSER method	UPLC-HRMS UHPLC-MS/MS QqQ	84	HRMS Method: 1.4–309.6 QqQ Method: 2–54	HRMS Method: 4.2–938.3 QqQ Method: Method: 5–163	[67]
Seafood (tuna, salmon, shrimp, tilapia, crab, cod, pollock, clam)	QuEChERS method	LC-MS/MS	44–160	<0.1 ^c^ 0.345 (PFBA), 207 (PFPeA)	N/A	[96]

**Table 6:** Summary of analytical procedures for the analysis of PFASs in honey and their recoveries (expressed as%), LODs (ng g^−1^), and LOQs (ng g^−1^).

Matrix	Extraction and Pretreatment	Analysis	Recoveries	LOD	LOQ	Ref.
Honey	QuEChERS method	micro-UHPLC-MS/MS	40–84 (PFOA) and 7–87 (PFOS)	0.016 (PFOA) and 0.040 (PFOS)	0.052 (PFOA) and 0.134 (PFOS)	[83]
Honey	QuEChERS method	micro-UHPLC-MS/MS	75–93	0.014–0.040	0.042–0.134	[109]

**Table 7:** Summary of analytical procedures for the analysis of PFASs in tap water, bottled drinking water, and beverages and their recoveries (expressed as%), LODs (ng L^−1^), and LOQs (ng L^−1^); N/A = not available; ^a^ = ng g^−1^; ^b^ = method detection limit (MDL); ^c^ = method quantification limit (MQL).

Matrix	Extraction and Pretreatment	Analysis	Recoveries	LOD	LOQ	Ref.
Tap and bottled water	ASE and SPE cleanup with Oasis WAX cartridge	UPLC-MS/MS	80.2–95.2	0.00008–0.00068 ^a^	0.00026–0.00225 ^a^	[114]
Tap and bottled water	Large volume direct injection (LVDI)	LC-MS/MS	87–114	0.05–1.0 ^b^	0.1–2.0 ^c^	[115]
Bottled water	SPE with Oasis HLB and Oasis WAX cartridges	LC-MS/MS	75–111	0.11–1.04 ^b^	N/A	[116]
Drinking water	DI	UHPLC-MS/MS	N/A	0.014–0.44 ^b^	0.030–1.0 ^c^	[111]
Drinking water	Offline SPE with Oasis WAX weak anion exchange cartridges	LC-MS/MS	66–138	0.28–18 ^b^	0.35–26 ^c^	[36]
Tap and bottled water	SPE with Oasis WAX cartridges	UHPLC-MS/MS	82–105	0.07–0.12 ^b^	N/A	[117]
Tap water	SPE with Oasis WAX cartridges	HPLC-MS	80–120	N/A	N/A	[118]
Bubble tea	USAE with ACN solvent followed by SPE cleanup with Oasis WAX cartridge	UHPLC-Orbitrap HRMS	43–114	0.001–0.012	0.002–0.057	[80]
Bottled water, juice, and tea	SPE with Presep PFC-II cartridges	LC-MS/MS	80.4–118.8	0.1–0.8	0.2–1.6	[113]
Tap water	Extraction with MeOH solvent followed by SPE cleanup with Oasis WAX SPE cartridge	UHPLC-MS/MS	97.6 (PFOA) and 106.2 (PFOS)	N/A	0.125–0.25	[100]
Tap water	SPE with Waters Oasis WAX cartridge	HPLC-MS/MS	60–125	0.01–0.13	0.01–0.47	[119]
Tap and bottles water	SPME procedure, HLB-WAX/PAN fiber	UHPLC-MS/MS	N/A	N/A	1–2.5	[110]
Juice	Extraction with MTBE solvent	LC-MS/MS	90.6–101.2 (PFOA) and 89.2–98.4 (PFOS)	0.038 ^a^ (PFOA) and 0.002 ^a^ (PFOS)	0.125 ^a^ (PFOA) and 0.007 ^a^ (PFOS)	[74]
Drinking water, tap water, and functional beverages (Mizone, vitamin water, and soda water)	MSPE procedure	HPLC-Orbitrap-HRMS	71.9–117.6	3–15	10–49	[82]

## Data Availability

The original contributions presented in the study are included in the article, further inquiries can be directed to the corresponding author.
